# Validating the Arabic Adolescent Nutrition Literacy Scale (ANLS): A Reliable Tool for Measuring Nutrition Literacy

**DOI:** 10.3390/nu17152457

**Published:** 2025-07-28

**Authors:** Sahar Obeid, Souheil Hallit, Feten Fekih-Romdhane, Yonna Sacre, Marie Hokayem, Ayoub Saeidi, Lamya Sabbah, Nikolaos Tzenios, Maha Hoteit

**Affiliations:** 1 Social and Education Sciences Department, School of Arts and Sciences, Lebanese American University, Byblos P.O. Box 36, Lebanon; saharobeid23@hotmail.com; 2 School of Medicine and Medical Sciences, Holy Spirit University of Kaslik, Jounieh P.O. Box 446, Lebanon; souheilhallit@usek.edu.lb; 3 Department of Psychology, College of Humanities, Effat University, Jeddah 21478, Saudi Arabia; 4 Applied Science Research Center, Applied Science Private University, Amman 11937, Jordan; 5 The Tunisian Center of Early Intervention in Psychosis, Department of Psychiatry “Ibn Omrane”, Razi Hospital, Manouba 2010, Tunisia; feten.fekih@gmail.com; 6 Faculty of Medicine of Tunis, Tunis El Manar University, Tunis 1007, Tunisia; 7 Department of Nutrition and Food Sciences, Faculty of Arts and Sciences, Holy Spirit University of Kaslik (USEK), Jounieh P.O. Box 446, Lebanon; yonnasacre@usek.edu.lb (Y.S.); mariehokayem@usek.edu.lb (M.H.); 8 Department of Physical Education and Sport Sciences, Faculty of Humanities and Social Sciences, University of Kurdistan, Sanandaj 66177-15175, Iran; a.saeidi@uok.ac.ir; 9 Faculty of Arts and Sciences, Modern University for Business and Science (MUBS), Damour P.O. Box 113-7501, Lebanon; lsabbah@mubs.edu.lb; 10 Center of Excellence in Research, Education, and Cultural Studies (CEREC), Beirut, Lebanon; 11 Faculty of Public Health, Charisma University, London EC1V 7QE, UK; 12 PHENOL Research Program, Faculty of Public Health, Section 1, Lebanese University, Beirut P.O. Box 6573, Lebanon; 13 Department of Primary Care and Population Health, University of Nicosia Medical School, Nicosia 2417, Cyprus; 14 INSPECT-LB (Institut National de Santé Publique, d’Épidémiologie Clinique et de Toxicologie-Liban), Beirut, Lebanon

**Keywords:** adolescent, nutrition literacy, scale, psychometric properties, Arabic

## Abstract

**Introduction**: Nutrition literacy has garnered growing research attention worldwide, yet only a few instruments have been developed to specifically measure this construct among adolescents. Accordingly, the present research sought to examine the validity and reliability of the Adolescent Nutrition Literacy Scale (ANLS) within a group of Lebanese adolescents. **Methods**: A cross-sectional study was carried out from December 2022 to March 2023, targeting a nationally representative sample. **Results**: Fit indices of the three-factor structure were good. Internal reliability was adequate for the following three subscales: Functional Nutrition Literacy (FNL) (ω = 0.88/α = 0.88), Interactive Nutrition Literacy (INL) (ω = 0.87/α = 0.86) and Critical Nutrition Literacy (CNL) (ω = 0.89/α = 0.89). Invariance was established across genders at configural, metric, and scalar levels. A significantly higher mean FNL and INL scores were found in males compared to females, with no significant difference between the two genders in terms of CNL. Higher FNL, but not CNL and INL scores were significantly associated with lower child food security. **Conclusions**: The Arabic ANLS has exhibited robust psychometric reliability, validity, and cost-effectiveness as a tool for assessing nutrition literacy. By utilizing the Arabic version of the ANLS, we can more efficiently and accurately assess the nutritional literacy of adolescents.

## 1. Introduction

Adolescence is a crucial stage for developing healthy habits that often persist into adulthood. Eating behaviors, a core part of lifestyle, are largely shaped during this period [1]. However, recent studies indicate that many adolescents are moving away from the traditional Mediterranean Diet (MD)—rich in plant-based foods, healthy fats, and complex carbohydrates– and are instead adopting a Western-style diet high in calories, ultra-processed foods, and saturated fats but low in essential nutrients [2,3,4]. In addition, about 80% of adolescents do not meet recommended physical activity levels, with sedentary behaviors such as excessive screen time being common [5,6]. Data from Mexico [7] reported that 36.3% of adolescents aged 12–19 years were overweight or obese, with higher rates observed among females. Recent evidence further links nutrition-related behaviors and issues in youth to their level of nutrition literacy [1,8,9].

Nutritional Literacy (NL) is a multidimensional construct that reflects an individual’s capacity to obtain, comprehend, interpret, and use nutrition-related information. It is commonly divided into three domains: functional, interactive, and critical nutrition literacy [10,11]. Functional Nutrition Literacy (FNL) refers to basic reading, writing, and numeracy skills needed to understand nutrition information. Interactive Nutrition Literacy (INL) involves the ability to communicate and actively engage with nutritional information, while Critical Nutrition Literacy (CNL) encompasses the skills to critically analyze and evaluate the reliability and relevance of such information [12]. As a fundamental, cost-effective, and practical tool, NL plays a vital role in promoting healthy nutrition and improving national health outcomes [9]. Consequently, investigating NL during adolescence is particularly important, as it can guide the development of sustainable nutrition interventions [11].

Appropriate nutritional literacy fosters healthier eating habits, enhances dietary quality, promotes better nutritional/food choices, and improves overall nutritional status. It also plays a critical role in preventing and managing nutrition-related non-communicable diseases [9,13,14,15]. These benefits are associated with factors such as improved nutrient adequacy, the ability to read food labels effectively, and greater food security [1,16,17]. Children with strong nutritional literacy are more inclined to develop healthy eating habits and make well-informed food choices, whereas low nutritional literacy has been linked to unhealthy dietary patterns [9,18,19]. Studies among adolescents have shown that nutrition literacy is linked to factors such as Body Mass Index, daily lifestyle practices, and overall eating behaviors [1]. Likewise, research has found that higher levels of nutrition literacy are associated with selecting smaller portion sizes when consuming fast food and consuming packaged or processed snacks less frequently among children and adolescents in school settings [20]. In the same way, a reciprocal relationship exists between food security and nutrition literacy, where limited food and nutrition literacy can contribute to food insecurity, and experiencing food insecurity may hinder the use of food literacy skills to maintain a nutritious diet [21].

Nutrition literacy has garnered growing research attention worldwide, yet only a few instruments have been specifically designed to assess this construct in children and adolescents. Most available tools focus primarily on adults [22,23,24,25]. A 2021 systematic review [26] identified four tools that measured NL or its subcomponents in children [26,27,28,29]. Of these, two addressed critical NL [27,28], one evaluated menu board literacy [29], and another focused on food label literacy [30]. Furthermore, in 2017, Asakura et al. developed the Nutrition Knowledge Questionnaire for primary school students. This tool comprises four sections that assess understanding of nutritional terms, knowledge of dietary guidelines, the application of information to food choices, and awareness of diet–disease relationships [31]. Another widely cited instrument is the Food and Nutrition Literacy (FNL) tool that evaluates both cognitive and practical skills of food and nutrition literacy in children [25]. However, these instruments often fall short in assessing deeper cognitive comprehension and critical evaluation of nutritional information. In China, Liao et al. created a nutrition literacy assessment for college students [32], while other researchers developed core NL items tailored for preschool-aged children [33,34]. It is important to note that cultural and social contexts strongly influence individuals’ beliefs and attitudes, thereby shaping their NL [35].

To better reflect the multifaceted nature of nutrition literacy, Bari (2012) developed the Adolescent Nutrition Literacy Scale (ANLS) [36], that was later on adapted into Turkish by Türkmen et al. [37]. It includes 22 attitude statements across three sub-dimensions [38]: Functional Nutrition Literacy (seven items); Interactive Nutrition Literacy (six items); and Critical Nutrition Literacy (nine items). The Cronbach’s alpha for internal consistency has been reported as 0.80 in its original development, indicating a good level of reliability. In studies adapting the scale to different languages and populations (e.g., Turkish), the Cronbach’s alpha increased to 0.86, further affirming its reliability in varied contexts. The Adolescent Nutrition Literacy Scale has been widely used in research to assess relationships between nutrition literacy and behaviors such as physical activity [39], dietary choices and health outcomes [40]. Thus, it is regarded as a reliable instrument for evaluating NL among adolescents.

Malnutrition remains a significant challenge in many Arab countries, highlighting the immense difficulty the region faces in achieving the 2030 Agenda for Sustainable Development goal of “zero hunger” and eliminating all forms of malnutrition [41]. The Arab region grapples with significant challenges related to food insecurity, malnutrition, and obesity, with an estimated 116 million people experiencing food insecurity, 43 million suffering from undernutrition, and 115 million obese [42]. Recent evidence shows a dramatic rise in malnutrition, with the number of undernourished individuals increasing from 4.8 million to 69 million between 2019 and 2020 [43].

In Lebanon, where the population faces the dual burden of macro- and micro-traumas, increasing their vulnerability to mental health disorders [44], recent studies highlight a concerning shift in adolescents’ dietary habits. This shift, characterized by the adoption of a Westernized lifestyle and greater sedentary behavior, has been linked to rising rates of overweight, obesity, and associated health complications [45,46,47]. These conditions often persist into adulthood, posing significant health challenges [48,49].

Moreover, with the rise of social media, the younger generation has become its primary user group. In Lebanon, this demographic is grappling with declining diet quality and increasing obesity rates [50]. Notably, adolescents are experiencing higher dietary fat consumption alongside reduced protein and carbohydrate intake, as well as the risk of insufficient mineral intake [51]. These trends may stem from a preference for fast food, frequent skipping of breakfast, and a general dislike of home cooking [52,53]. While the immediate health effects may not always be evident, these unhealthy lifestyles and dietary patterns significantly heighten the long-term risk of chronic diseases [54].

Addressing this issue requires urgent efforts to improve adolescent’s nutritional literacy through targeted education [55]. Before implementing specialized nutritional care and educational programs for specific groups, it is essential to thoroughly understand their perceived nutritional literacy. This study focuses on culturally adapting the ANLS. We hypothesized that the Adolescent Nutrition Literacy Scale would (1) show three factors similar to the original version, (2) exhibit robust psychometric validity and reliability and invariance between males vs. females, and (3) correlate with child food security.

## 2. Methods

### 2.1. Human Ethics and Consent to Participate Declarations

The study was carried out according to the ethical principles of the Helsinki Declaration (2013 version). The Ethics Committee of the Al-Zahraa University Medical Center, Beirut, Lebanon (Reference # 10-12-2022) provided their ethical approval to conduct this study. Informed written consent was obtained from all participants prior to their inclusion in the study.

### 2.2. Study Design

**Figure 1:**
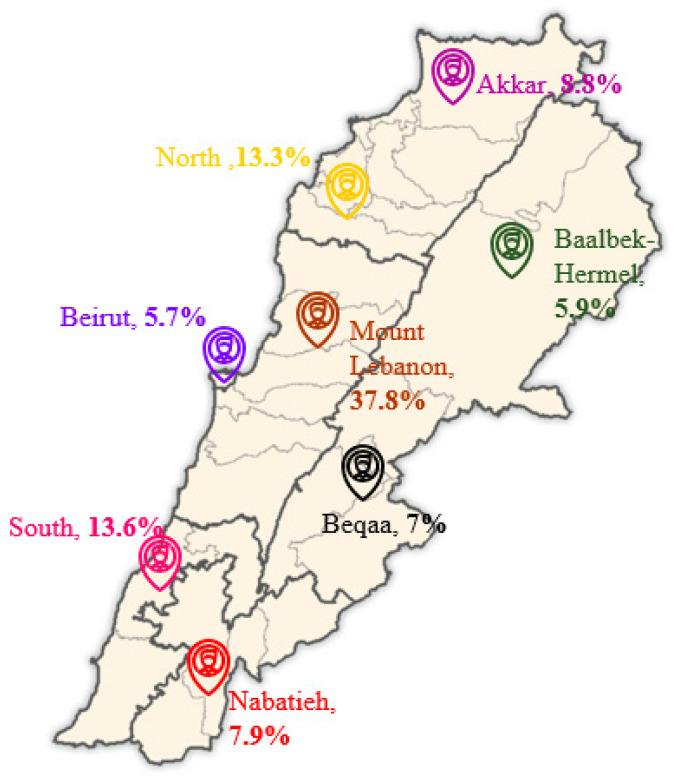
Distribution of study participants across governorates [56].

A cross-sectional survey was carried out between 11 December 2022 and 18 March 2023, targeting a nationally representative sample of Lebanese adolescents. Participants were selected through a probability cluster sampling approach and recruited from all eight Lebanese governorates. Participants’ distribution across the governorates is shown in Figure 1. To ensure relevance to the goals of the study, participants were recruited based on the following eligibility criteria: All the participants had to be Lebanese, aged between 10 and 18 years, and free of chronic diseases. Moreover, from each household, only one adolescent child was recruited after dissemination of the survey announcement in several public areas, social media, and healthcare settings.

### 2.3. Data Collection and Measures

Using a pre-tested questionnaire, the following information was collected during a face-to-face interview with the participants: age, gender, residency, primary caregiver, whether currently working, education level, school type, and whether the adolescent receives nutrition education in their schools. Information regarding nutrition literacy was collected during the same interview where the Adolescent Nutrition Literacy Scale (ANLS) was utilized to assess the nutrition literacy levels of adolescents. Originally developed by Bari [36], the scale was forward and backward translated to Arabic by two different translators. The original and translated English versions were then compared by the research team and the two translators to solve any discrepancies. A pilot study was conducted on 30 adolescents to make sure that all questions were clear to them; no changes were made afterwards. The ANLS comprises 22 items, each rated on a five-point Likert scale, where 1 = strongly disagree, 2 = disagree, 3 = undecided, 4 = agree, and 5 = strongly agree. Scores on the scale range from a minimum of 22 to a maximum of 110. A score between 22 and 57.2 reflects “low nutrition literacy”, 57.2 to 74.8 indicates “moderate nutrition literacy”, and 74.8 to 110 represents “high nutrition literacy”.

Child food security: The child questionnaire consists of 14 items designed to assess food insecurity experiences among children. It has been previously validated in Arabic and refined based on prior research [57]. The 14 items are organized into five key domains: cognitive, emotional, and physical awareness of food scarcity, coping strategies, and shared responsibility with caregivers for managing resources. The questionnaire covers experiences from the past 6 months, specifically from the end of the last academic year to the beginning of the summer. Participants respond with options of “sometimes” (a little of the time), “often” (a lot of the time), or “never.” In this study, the Cronbach’s α = 0.94.

### 2.4. Analytic Strategy

A confirmatory factor analysis (CFA) was performed using SPSS AMOS v.28. The required sample size was estimated at a minimum of 440 participants, following the guideline of 20 times per variable of the scale [58]. Parameter estimates were obtained through the maximum likelihood method. Several fit indices were computed to evaluate model fit [59], including Root Mean Square Error of Approximation (RMSEA; ≤0.08), Standardized Root Mean Square Residual (SRMR; ≤0.05), Tucker–Lewis Index (TLI; ≥0.90) and Comparative Fit Index (CFI; ≥0.90). Convergent validity was assessed through the average variance extracted (AVE), with values of ≥0.50 considered acceptable [60]. Since multivariate normality was initially not met (Bollen-Stine bootstrap *p* = 0.002), a non-parametric bootstrapping procedure was applied.

A multi-group CFA was conducted to test measurement invariance of ANLS scores across genders [61] at the configural, metric, and scalar levels [62]. Evidence of invariance was supported when ΔCFI was ≤0.010 and ΔRMSEA was ≤0.015 or ΔSRMR was ≤0.010 [63]. Gender differences in ANLS scores were examined using the Mann–Whitney test.

Internal reliability was evaluated through McDonald’s ω and Cronbach’s α, with values above 0.70 indicating satisfactory reliability [64]. Associations between ANLS and child food security scores were analyzed using Spearman’s correlation test.

## 3. Results

Descriptive statistics of the sample can be found in Table 1.

### 3.1. Confirmatory Factor Analysis

**Figure 2:**
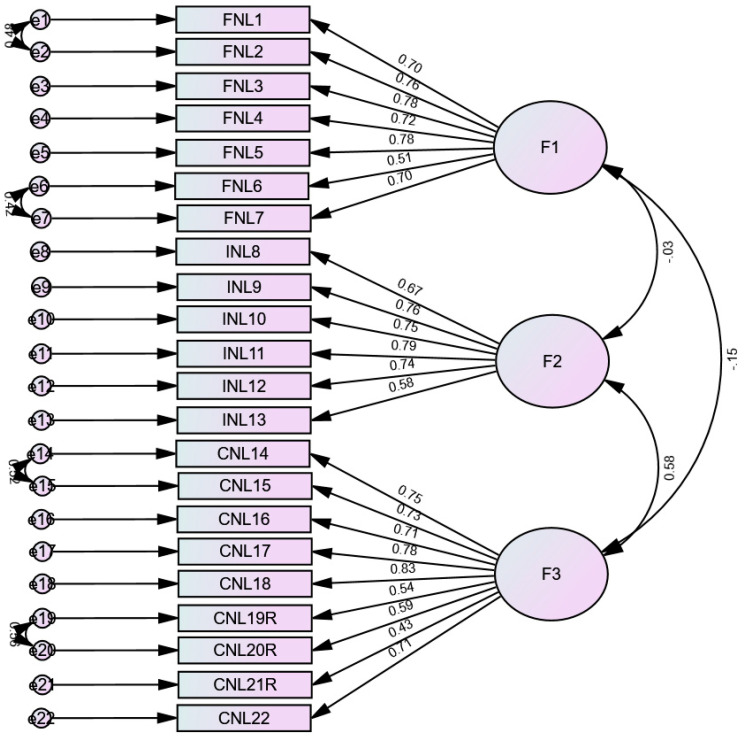
Standardized loading factors deriving from the confirmatory factor analysis of the Adolescent Nutrition Literacy Scale in Arabic. F1 = Functional Nutrition Literacy; F2 = Interactive Nutrition Literacy; F3 = Critical Nutrition Literacy.

The fit indices were good (RMSEA = 0.066 (90% CI 0.060, 0.072), SRMR = 0.062, CFI = 0.924, TLI = 0.913). The standardized estimates of factor loadings were all adequate (Figure 2). Internal reliability was adequate for the three subscales: Functional Nutrition Literacy (ω = 0.88/α = 0.88), F2 = Interactive Nutrition Literacy (ω = 0.87/α = 0.86), and F3 = Critical Nutrition Literacy (ω = 0.89/α = 0.89).

### 3.2. Gender Invariance

Invariance was shown at the metric and scalar levels in terms of genders (Table 2). A significantly higher mean FNL (23.22 ± 5.64 vs. 21.45 ± 6.00; *p* = 0.001, Cohen’s d = 0.304) and INL (18.20 ± 4.88 vs. 17.07 ± 5.45; *p* = 0.018, Cohen’s d = 0.220) were found in males compared to females, with no significant difference between the two genders in terms of CNL (30.41 ± 6.92 vs. 29.72 ± 7.33; *p* = 0.161, Cohen’s d = 0.096).

### 3.3. Concurrent Validity

Higher FNL (r = −0.15; *p* = 0.002), but not CNL (r = −0.06; *p* = 0.196) and INL (r = 0.001; *p* = 0.981), scores were significantly associated with lower child food security.

## 4. Discussion

This study’s findings demonstrate that the ANLS is a reliable and valid instrument for assessing nutrition literacy in adolescents, making it a valuable tool for nutritional monitoring. The results further affirm its reliability, validity, and suitability for use among Arabic-speaking adolescents.

### 4.1. Factor Structure

Regarding the factorial validity of the Arabic version of the ANLS, our findings are consistent with the original study [36] and the Turkish validation [37], confirming that the scale includes three sub-categories: Functional, Interactive, and Critical nutrition literacy. These dimensions highlight the complex nature of nutrition literacy and emphasize the need for tailored educational interventions. The factor structure of the ANLS offers a strong framework for evaluating nutrition literacy among adolescents [37]. By identifying the distinct dimensions—functional, interactive, and critical nutrition literacy—the scale not only assesses adolescents’ current abilities but also informs targeted interventions aimed at fostering healthier lifestyles. Future research should continue to validate the ANLS in diverse populations and examine its potential to predict long-term dietary behaviors and health outcomes.

### 4.2. Internal Reliability

In our study, the reliability coefficients were very good: Functional Nutrition Literacy (ω = 0.88/α = 0.88), Interactive Nutrition Literacy (ω = 0.87/α = 0.86), and Critical Nutrition Literacy (ω = 0.89/α = 0.89), with higher values than the Turkish validation [37]. Cronbach’s alpha values were 0.66 for FNL, 0.71 for INL, 0.84 for CNL, and 0.80 for the total score obtained in the tool. Similarly, in the scale development study [36], the internal consistency coefficient was reported to be 0.80 for the overall scale. Subscales exhibited reliability values ranging from 0.648 to 0.942, indicating acceptable to excellent internal consistency across different dimensions of nutrition literacy. Accordingly, based on the findings of this research, the Arabic ANLS appears to exhibit robust psychometric reliability, indicating that this tool measures nutrition literacy with an acceptable level of accuracy.

### 4.3. Sex Invariance

In this study, notable differences were observed between males and females in ANLS scores, with higher mean FNL and INL in males compared to females, and with no significant difference between the two genders in terms of CNL. It is worth noting that the developmental scale and Turkish validation do not report gender invariance, highlighting the originality of our findings.

A study conducted across 10 Arab countries [40] reported that female adolescents had higher nutritional literacy than males. The same results were found in two Turkish [1,65] and two Iranian [17,66] studies. One possible explanation for these gender differences is that females tend to prioritize healthy eating and focus more on the nutritional value of food [67]. Women often have greater nutrition knowledge and view nutrition as an integral component of their overall health [68].

Regarding CNL, a previous study [69] found that a higher proportion of women (60%) than men (42%) struggled to differentiate between scientific and non-scientific dietary information. Additionally, women were more likely than men to be influenced by dietary advice in the media (42% vs. 27%) and to find alternative medicine advice credible (38% vs. 25%) [69]. In contrast, our findings suggest no significant gender differences in CNL, possibly indicating that both genders face similar challenges in critically assessing complex nutritional information. Research suggests that men and women alike may struggle to discern credible from non-credible nutritional advice, underscoring a universal need to improve critical thinking skills in this area [40].

One possible explanation for the higher scores in FNL and INL among males in this study may relate to contextual factors such as demographic characteristics, educational level, or evolving health behaviors. Recent evidence suggests that men have become increasingly engaged in health and nutrition topics, especially in populations where health promotion campaigns and fitness culture are widespread [70]. Cultural norms and media exposure may also influence gender-specific information-seeking behavior, with some studies showing that men are more likely to seek out functional health information when it aligns with their personal goals, such as physical performance or body image [71]. Differences in sampling methods and measurement tools across studies may further account for discrepancies in findings [71].

These findings emphasize the importance of developing gender-sensitive nutrition education strategies that address specific gaps while leveraging strengths across different literacy domains.

### 4.4. Concurrent Validity

In the current study, higher FNL, but not CNL and INL, scores were significantly associated with lower child food security, in line with many studies demonstrating a strong link between high food insecurity and unfavorable eating behaviors, which ultimately lead to poor dietary quality [72,73,74]. For instance, research involving children aged nine to twelve found that those experiencing high food insecurity exhibited lower levels of nutritional knowledge and struggled to comprehend food and nutrition information [75]. Additionally, their food choice literacy scores were significantly lower compared to their food-secure peers. Moreover, Landry et al. reported that food-insecure children had poorer diet quality, scoring lower on greens and beans, seafood, and plant proteins, while consuming more added sugars compared to their food-secure peers [72]. Likewise, a study of 3790 food-insufficient, low-income families found that adolescents consumed fewer calories, carbohydrates, and fruits, but had higher cholesterol intake [76]. Moreover, adolescents experiencing food insecurity often shop at stores dominated by less-healthy options and are more likely to choose unhealthy snacks and sugar-sweetened beverages [77]. While food literacy—an essential factor in shaping eating behaviors—can help food-insecure adolescents improve their food choices by building critical skills, several unmodifiable factors contribute to poor dietary decisions. Contributing factors include household income, restricted availability of healthy foods, and the elevated cost of nutritious choices.

### 4.5. Clinical Implications

Developing a valid Arabic version of the ANLS would enable the collection of precise epidemiologic data on nutrition literacy across Arab countries. This, in turn, could guide initiatives to enhance nutrition literacy among adolescents, a critical step in fostering healthy eating habits and lifestyle behaviors. Raising adolescents’ awareness is essential, and health policies should prioritize the development of appropriate educational resources to support this objective. Moreover, Arab schools can present an excellent opportunity to integrate efforts toward sustainable development by leveraging the formal education system to enhance students’ nutrition literacy [78]. All school-based activities promoting healthy eating—both inside and outside the classroom—can form part of a broader nutrition education strategy known as a “macro-curriculum” [79].

### 4.6. Limitations

When interpreting the results and conclusions of this study, several limitations must be considered. First, the use of snowball sampling for participant recruitment may limit the generalizability of the findings. In the context of Lebanon where private schools often serve students with higher socioeconomic backgrounds, the overrepresentation of children from private schools (almost twice as many as from public schools) could have influenced the results. Second, cross-sectional design restricts our ability to assess certain psychometric properties, such as test–retest reliability. Third, key psychometric properties, including inter-rater and test–retest reliability, and discriminant and convergent validity, were not evaluated. Fourth, the reliance on self-reported data from participants introduces the potential for information bias. Fifth, as the sample was drawn from the Lebanese population, the findings may not be applicable to all Arabic-speaking countries. Further research is needed to explore cultural differences across the Arabic-speaking world. The face-to-face data collection may have introduced social desirability bias, and the lack of a separate validation sample limits generalizability.

## 5. Conclusions

The Arabic Adolescent Nutrition Literacy Scale (ANLS) has exhibited robust psychometric reliability, validity, and cost-effectiveness as a tool for assessing nutrition literacy. The adapted and validated version of the ANLS is well suited for Arabic-speaking adolescents, and its potential for use in other countries warrants further investigation. By utilizing the Arabic version of the ANLS, we can more efficiently and accurately assess the nutritional literacy of adolescents. This, in turn, facilitates the development of targeted nutrition education programs and public health policies. Additionally, it allows for the identification of high-risk groups, enabling the provision of necessary support and interventions to enhance the overall nutritional health of society.

## Figures and Tables

**Table 1:** Sociodemographic and other characteristics of the sample (N = 442).

Variable	N (%)
Sex	
Male	246 (55.7%)
Female	196 (44.3%)
Education level	
Primary	112 (25.3%)
Complementary	119 (26.9%)
Secondary	122 (27.6%)
University	89 (20.1%)
School type	
I am currently not attending school	51 (11.6%)
Public school	168 (38.1%)
Private school	222 (50.3%)
	Mean ± SD
Age (years)	14.66 ± 2.94

**Table 2:** Measurement invariance of the Adolescent Nutrition Literacy Scale across genders.

Model	CFI	RMSEA	SRMR	Model Comparison	ΔCFI	ΔRMSEA	ΔSRMR
Males	0.918	0.067	0.060				
Females	0.893	0.084	0.084				
Configural	0.906	0.053	0.060		0.002	0.002	<0.001
Metric	0.908	0.051	0.060	Configural vs. metric	0.002	0.002	<0.001
Scalar	0.906	0.051	0.060	Metric vs. scalar	0.002	<0.001	<0.001

Note. CFI = Comparative fit index; RMSEA = root mean square error of approximation; SRMR = standardized root mean square residual.

## Data Availability

Data is provided within the manuscript.
